# Can plant traits predict seed dispersal probability via red deer guts, fur, and hooves?

**DOI:** 10.1002/ece3.5512

**Published:** 2019-08-13

**Authors:** Tanja K. Petersen, Hans Henrik Bruun

**Affiliations:** ^1^ Department of Natural History, NTNU University Museum Norwegian University of Science and Technology Trondheim Norway; ^2^ Centre for Biodiversity Dynamics NTNU Trondheim Norway; ^3^ Department of Biology University of Copenhagen Copenhagen Denmark

**Keywords:** *Cervus elaphus*, dispersal mode, endozoochory, epizoochory, seed dispersal, seed functional traits

## Abstract

**Abstract:**

Seed dispersal by mammals provides functional connectivity between isolated plant habitat patches. Across much of Europe, red deer (*Cervus elaphus*) populations are growing steadily, potentially leading to increasing importance of this large mammal species to plant dispersal. While deer endozoochory is relatively well studied, epizoochory via fur and hoof attachment is much less understood. Seed dispersal internally and externally on 57 red deer individuals was investigated by sampling the seed content of intestinal tracts, fur, and hooves of animals shot during annual hunts in four contrasted landscapes in Denmark. We assessed compositional differences between dispersal modes whether plant species' association to a dispersal mode could be predicted by seed traits, whole‐plant traits, and species' local abundance. We found the largest difference in seed species composition to be between epizoochory (fur and hooves) and endozoochory (gut contents). Probability of plant dispersal through guts and fur was correctly predicted from traits more often than not. Hoof‐epizoochory, however, could not be correctly predicted from plant traits. Most plant species encountered were picked up by all three dispersal modes, suggesting an overriding effect of plant abundance in the landscapes in which the deer roam, which was also indicated by the statistical analysis. Nonetheless, a significant proportion of species were associated with either gut, fur, or hoof‐borne dispersal, reflecting the effect of plant traits and, potentially, animal behavior. Plant species being dispersed more often than expected through intestines were mainly associated with ruderal habitats, whereas species transported via fur tended toward association with wooded habitats. Considering the increasing red deer populations in Europe, and the differences between seed dispersal modes, all modes of animal seed dispersal should be taken into account in future studies.

**OPEN RESEARCH BADGES:**

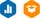
This article has been awarded Open Data and Open Materials Badges. All materials and data are publicly accessible via the Open Science Framework at https://doi.org/10.6084/m9.figshare.7982483 and https://doi.org/10.6084/m9.figshare.7982483

## 1 INTRODUCTION

Large herbivores have key impacts on vegetation structure and species composition of plant communities (Burns, Collins, & Smith, 2009; Ripple et al., 2015). Through mechanical disturbance of biomass, nutrient deposition, and modulation of interspecific competition between plants, animals can shape habitat conditions for plant species on the landscape scale (Augustine & McNaughton, 1998). However, the engineering role of large herbivores is not limited to light conditions and competitive hierarchies between plant species. They may also transfer diaspores to unoccupied suitable habitat and promote local establishment by soil disturbance (Iravani et al., 2011; von Oheimb, Schmidt, Kriebitzsch, & Ellenberg, 2005; Picard & Baltzinger, 2012; Picard, Chevalier, Barrier, Boscardin, & Baltzinger, 2016). This may be especially important in fragmented landscapes (Graae, 2002; Panter & Dolman, 2012).

Zoochory is mediated by different means. For herbivorous megafauna, endozoochory goes via ingestion of diaspores with foliage and subsequent defecation or regurgitation of viable propagules (Baltzinger, Karimi, & Shukla, 2019; Janzen, 1984). Epizoochory is most often taken to mean transport of (viable) diaspores attached to fur or plumage (Albert, Auffret, et al., 2015). However, diaspores are also transported while attached to the feet of animals. The latter process has rarely been investigated in any detail (but see reviews in Albert, Auffret, et al., 2015 and Baltzinger et al., 2019).

Ungulates include some of the largest, native herbivores in Northern Europe, and one of the most widespread species is the red deer (*Cervus elaphus*; Burbaitė & Csányi, 2010). Red deer has important ecological effects on vegetation structure, mainly through grazing, browsing, bark stripping, trampling, and dispersal of plant seeds (Baltzinger et al., 2019; Mysterud, 2006; Olff & Ritchie, 1998). Over vast tracts of Europe, red deer populations have multiplied during the latest decades (Burbaitė & Csányi, 2010; Milner et al., 2006). Thus, the effects and the extent of potential seed dispersal are highly interesting, not least from a conservation perspective.

The probability for plant species to have diaspores transported through red deer intestines, fur, and/or hooves is probably dependent on (a) plant species abundance in the landscape (Bruun & Poschlod, 2006; Karimi, Hemami, Tarkesh Esfahani, Akhani, & Baltzinger, 2018; Picard et al., 2016), (b) whole‐plant traits, such as diaspore number per ramet and diaspore release height (Albert, Auffret, et al., 2015; Albert, Mårell, Picard, & Baltzinger, 2015), and (c) diaspore traits, such as presence of specific appendages promoting attachment (Lepková, Horčičková, & Vojta, 2018). Behavior, including feeding preferences, is also likely to affect deer–plant interactions (Liehrmann et al., 2018). Thus, focussing on groups of plant species sharing certain traits, rather than on individual species, may reach a higher level of generalization. Moreover, endozoochory and epizoochory can be complementary dispersal mechanisms, effective for different portions of the regional plant species pool (Couvreur, Cosyns, Hermy, & Hoffmann, 2005), but depending on the characteristics of the vector (Baltzinger et al., 2019). For all dispersal modes, the number of diaspores available for contact with dispersal agents such as deer is crucial. Since seed number per ramet is traded off against seed size, per unit reproductive effort, seed size seed may to a large degree be used as a proxy for seed output per plant (Bruun & Poschlod, 2006). However, seed size may also be related to susceptibility to being crushed in the molar mill and resistance to digestive fluids. Albert, Mårell, et al. (2015) showed seed shape (deviation from sphericity) to be related to dispersal route as well. To take these points into account, we used seed number per ramet, seed shape, and seed mass as key traits in predicting dispersal probability.

The release height of the diaspores can affect the attachment and retention potential of diaspores in the fur, with seeds presented near the soil surface being less likely to becoming attached to favorable positions on the animal, such as the back (Albert, Auffret, et al., 2015; Graae, 2002; Wessels, Eichberg, Storm, & Schwabe, 2008). It may also affect endozoochory, as plant height in general influences in probability of ingestion by both mixed feeders and concentrate selectors (Albert, Mårell, et al., 2015).

Possession of adhesive appendages, such as hooks and bristles, has been shown to increase the attachment and retention potential of individual diaspores (Couvreur, Couvreur, Vandenberghe, Verheyen, & Hermy, 2004; Graae, 2002; Kiviniemi, 1996). The positive effect of appendages on adhesion/retention in fur is nonetheless often overridden by a negative effect of diaspore mass (heavier seeds are more likely to drop off quickly), as appendages are associated within larger diaspores across plant species (Tackenberg, Römermann, Thompson, & Poschlod, 2006). Small and smooth seeds may penetrate into the fur coat and get transported over vast distances (Albert, Mårell, et al., 2015; Fischer, Poschlod, & Beinlich, 1996; Römermann, Tackenberg, & Poschlod, 2005).

The few existing studies of hoof‐borne seed dispersal have found no obvious predictors for attachment or retention potential (Heinken & Raudnitschka, 2002), but see Albert, Auffret, et al. (2015) for a review.

Habitat association of plant species can be associated with dispersal traits. In particular, plant species found in spatially unpredictable patchy habitats, such as heavily disturbed sites, may rely more on dispersal in space than species typical of the landscape matrix. Therefore, we used at broad classification of preferred habitat by plant species as a predictor for seed dispersal via guts, fur, and hooves, similarly to the other included plant traits.

Our aim was to assess the differences between red deer endozoochory, fur‐epizoochory, and hoof‐epizoochory in terms of plant species composition and in terms of dispersal‐related plant traits. Specifically, we ask: (a) Does seed species composition differ between the three deer‐mediated seed dispersal routes (guts, fur, hooves)? (b) Can seed traits and whole‐plant traits predict the dispersal route preferentially taken by plant species?

## 2 MATERIALS AND METHODS

### 2.1 Study sites

**Figure 1:**
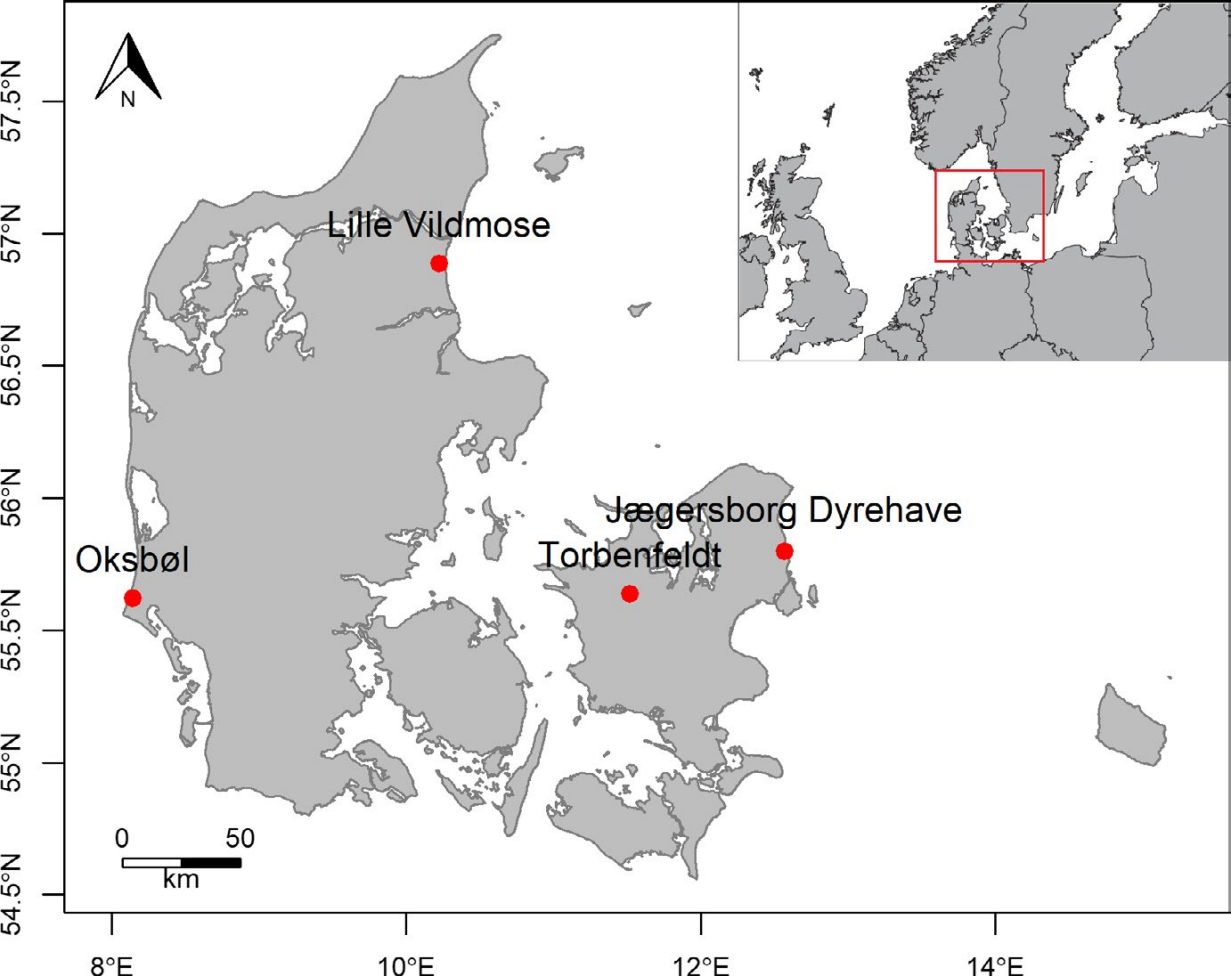
Sampling locations within Denmark. Red deer were sampled during annual hunts in September‐December 2015. In total, 57 red deer were sampled: 10 from Jægersborg Dyrehave (sampled on 22 September, 20 October, and 28 October), 21 from Lille Vildmose (sampled on 9–14 October and 2 November), 22 from Oksbøl (sampled on 29 October, 3 November, 11 November, and 10 December) and 4 from Torbenfeldt (sampled on 27 November). Inset map shows the location of Denmark within north‐western Europe

The study was conducted in four areas in Denmark during the hunting season (September–December) of 2015: Oksbøl (approx. 2,745 ha, sampled on 29 October, 3 November, 12 November, and 10 December) and Lille Vildmose (approx. 3,993 ha, sampled on 9–14 October and 2 November) in Jutland, Torbenfeldt (approx. 1,632 ha, sampled on 27 November) and Jægersborg Dyrehave/Deer Park (approx. 1,100 ha, sampled on 22 September, 20 October, and 28 October) in Zealand (Figure 1). Mean annual temperature and mean yearly precipitation in Denmark are 8.3°C and 746 mm (DMI, 2019), respectively. Differences between the sampling sites are small. The areas differed in predominant land‐use and regional plant species pool. Lille Vildmose and Jægersborg Dyrehave are large fenced reserves, while Oksbøl and Torbenfeldt are not fenced. The Lille Vildmose and Oksbøl areas are dominated by mixed forests, plantations, and dwarf shrub vegetation (heathland and raised bog) on sandy or peaty soils, while the Torbenfeldt and Jægersborg Dyrehave areas are situated on richer, more clayey soils with deciduous forests and either farmland or grasslands as the main component. Supplemental feeding is practiced at all sites to some degree, but is very substantial at Torbenfeldt and Jægersborg Dyrehave in particular.

In total, 57 red deer were sampled: 10 from Jægersborg Dyrehave, 21 from Lille Vildmose, 22 from Oksbøl, and 4 from Torbenfeldt. All samples were taken from newly shot deer during the yearly regulatory hunts, in most cases on the spot where the animal fell. In a few cases, the dead animal was moved prior to sampling, but dragging through vegetation was always kept at a minimum in order to avoid contamination. All samples were collected by the same person, ensuring consistency in the methodology.

### 2.2 Diaspores in fur and hooves

After the deer were shot, the fur was brushed, first with a regular metal comb (tooth space: 2 mm) and then with a metal louse comb (tooth space: 0.3 mm). In order to retrieve all adhering diaspores, fur on the entire upwards‐facing side of the dead animal was combed thoroughly, including legs and head‐region. Hooves were brushed thoroughly with a toothbrush and mud and debris collected, on all sides, underneath and between the phalanges. Combings and brushings were investigated under a dissection microscope and diaspores identified based on morphological characters using specialized literature (Anderberg, 1994; Berggren, 1969, 1981; Cappers, Bekker, & Jans, 2006; Grigas, 1986).

### 2.3 Diaspores in gut contents

After the animal was cut open, fecal material was taken from the distal part of the intestine, placed in paper bags, and dried at 25–30°C for 10 days, and subsequently subjected to dry, cold stratification at 1°C for 6 weeks. For six animals, no fecal material was present in the gut. As it was important for the study to match fecal samples with fur and hoof samples, it was not desirable to collect fecal samples from the ground. To discriminate between more traditional sampling from the ground and the methodology used here, we will refer to the samples of gut content as “gut samples.” The entire gut sample (ranging from 0.18 to 80.2 g) was then soaked in water, gently crushed and mixed with potting soil (1:1), and spread in trays on top of a mixture of potting soil, vermiculite, and perlite (50:25:25 volume ratio) in a layer no thicker than a maximum of 1 cm. Trays were kept in a greenhouse at approximately 25°C with 15 hr of artificial light and 9 hr of darkness during winter and spring 2015/2016 (December–June). Six control trays, only containing potting soil and vermiculite/perlite/potting soil mixture, were included. Trays were shuffled on the greenhouse bench every week. As soon as seedlings could be identified, they were removed to minimize competition and to promote additional seed germination. The germination experiment was done to ensure that the transported diaspores were viable.

Three diaspores could not be identified to species level at all (one seed belonging to the family Asteraceae and two seeds being either the genus *Cirsium* or the genus *Carduus*) and were not included in the analyses. As seedlings from *Carex disticha* and *Carex arenaria* could not be distinguished, the traits of only one of the species were accounted for. The reported species numbers are an absolute minimum of species, assuming that all unidentified species belong to an already identified species (unidentified species that clearly did not belong to an identified species are exceptions to this statement, e.g., *Poa* sp. that clearly did not belong to either *Poa annua* or *Poa trivialis*).

### 2.4 Data analysis

All statistical analyses were made in R, version 3.4.1 (R Core Team, 2018).

Differences in diaspore numbers and species numbers among dispersal modes were evaluated using GLMM with animal identity nested within sampling site as random factor and Poisson errors, differences subsequently compared by multiple comparisons of means using Tukey Contrasts (function glht in the package multcomp; Hothorn et al., 2019).

In order to assess overall differences in plant species composition between dispersal modes, a species‐by‐sample matrix was subjected to Nonmetric Multidimensional Scaling (NMDS) with Bray–Curtis dissimilarity index and other default settings of the metaMDS function in the vegan‐package (Oksanen et al., 2016). Samples scores on the first three NMDS ordination axes were then compared in linear mixed effects models (LMM) as implemented in the lmer‐function of the package lme4 (Bates, Mächler, Bolker, & Walker, 2015) and using the individual animal identity nested in sampling site as random effects. Separate models were made for each NMDS axis. The models were compared to the null model (only including random effects) with the function drop1(), comparing AIC values. For the NMDS, none of the identified taxa were pooled. As the number of unidentified individual diaspores/seedlings and species was quite small relative to the total numbers, their statistical impact was evaluated to be minor. Four dimensions were used in the ordination, three significant dimensions and a fourth dimension to account for noise. Three sampled animals were excluded from the analysis, as they proved to be so vastly different from the rest that they obscured all other underlying patterns. The omitted samples contained either (a) only a single seed, which was not observed in any other samples (*Myosotis laxa*), (b) several diaspores of only one species, which was observed in only one other sample (*Juncus tenuis*), or (c) only a single seedling of a species that was well represented in other samples (*Sagina procumbens*).

Dispersal‐related whole‐plant and diaspore traits were retrieved from the Ecoflora (Fitter & Peat, 1994) and LEDA databases (Kleyer et al., 2008), using the *TR8*‐package (Bocci, 2015). We used mean seed mass, diaspore release height, and number of diaspores per ramet (“seed number per plant” = SNP). For SNP, only records for “per ramet/tussock or individual plant” were included. We also included seed shape (variance in dimensions, Vs Thompson, Band, & Hodgson, 1993), with data on seed length, breadth, and depth per plant species obtained from Grigas (1986). To take into account the effect of plant species' abundance in the landscape, a measure of species' frequency was obtained from 5 km × 5 km grid cell data available from all four study locations (Hartvig & Vestergaard, 2015). The per species occupancy (presence/absence) in 25 grid cells (25 × 25 km^2^) centered on each sampling location was used as a proxy for abundance (referred to as “landscape occupancy” in the following). Values range between 0 (not present in any of the grid cells; a hypothetical value) and 1 (present in all grid cells). Although these data were collected for a different purpose and the inherent abundance scale (1–25 grid cells occupied on a total area of 25 × 25 km^2^) had very low abundance resolution among common species, we take a signal from this landscape occupancy measure in predictions as a strong indication that species' abundance matters.

Habitat associations of each species were categorized into grassland (G) (including semi‐natural open habitat, both dry, damp, and heathland), wetland (We) (including mires, both open and shrubby), woodland/forest habitats (Wo), and arable/ruderal habitats (R) (including species growing in a variety of disturbed habitats, such as spoil, quarries, and wastelands, based on Grime, Hodgson, and Hunt (2007) and Frederiksen, Rasmussen, and Seberg (2012)). W and G‐species were separated based on their Ellenberg Moisture value stated in Ecoflora or from Pignatti, Menegoni, and Pietrosanti (2005); species with Ellenberg Moisture value ≥8 were scored as wetland species (Grime et al., 2007; Pignatti et al., 2005).

The diaspore appendage trait was classified as “No appendages,” “Hairs,” “Hooked,” “Winged,” “Bristly,” or “Other” (anything that could not be assigned to any of the other five categories), based on visual inspection. If more than one type of appendages were present, the most prominent one was used for categorization.

Influence of dispersal traits on dispersal mode was assessed with a linear discriminant analysis (LDA) of seed mass, release height and seed number (all log‐transformed), seed shape, and landscape occupancy, using the lda function in the MASS package (Venables, & Ripley, 2002). The variables were standardized using the scale function, to allow for subsequent comparison of coefficients. The data set was randomly split, using 50% of the data to build the LDA model. The LDA model was evaluated by assessing the misclassification rate for prediction of dispersal mode for the observed plant species of the remaining 50% of the data, and relative variable importance assessed by comparing the absolute values of the discriminant coefficients. The analysis was based on the individual diaspores to account for differences in frequency between the different species.

Habitat association and appendages were analyzed with a Fisher's exact test. Here, only data on whether the species was dispersed were included, not the number of diaspores, as overly abundant species would have masked potential patterns. The number of dispersed species within each category was tested against the null hypothesis of no difference between categories. To determine the likely drivers of a possible dependence, the Pearson residuals from a *Χ* ^2^‐distribution were evaluated.

## 3 RESULTS

A total of 4,616 diaspores and 958 seedlings were identified in the epi‐ and endozoochorous samples, respectively. The diaspores belonged to 66 plant species in total (conservative count; Table 1; full species lists in Appendix, Table A1 and Table A2). In Table A1, taxa only identified to genus or a less specific taxonomic level are presented as separate species to give a full presentation of the results. However, in most analyses the conservative approach described previously was followed.

**Table 1:** Numbers of identified species and diaspores for each site and in total. The samples were collected on red deer shot during the annual regulatory hunts in September–December 2015. The species numbers are conservative, assuming unidentified species to belong to an already identified species (unless this was obviously not the case). The number of species and diaspores are the sum across all sampled individual deer within a site, within each dispersal mode

Site	Fur		Hooves		Feces		Total	
	Species	Diaspores	Species	Diaspores	Species	Diaspores	Species	Diaspores
Jægersborg Dyrehave (*n* = 10)	10	699	10	321	15	438	24	1,458
Lille Vildmose (*n* = 21)	17	2,894	21	336	26	420	44	3,650
Oksbøl (*n* = 22)	13	251	9	48	9	97	21	396
Torbenfeldt (*n* = 4)	7	39	7	28	2	3	11	70
Total	32	3,883	32	733	33	958	65	5,574

For both fur‐ and hoof‐epizoochory, the nationally most common species is Common rush (*Juncus effusus*), presenting, respectively, 76% and 49% of all diaspores.

In total, across all dispersal modes, 18 species (27% of all species) were found only once (as a single diaspore), and 42 species ( 64%) were found only at one site.

Germination occurred in 84% of the gut samples (43 out of 51 samples), with a mean seedling density of 108.5 seedlings per 100 g feces. A total of 958 seedlings from 33 species germinated. No seedlings emerged from the control trays; hence, all emerged seedlings can be assumed to be of endozoochorous origin.

The nationally most common species is Common bent (*Agrostis capillaris*; 19% of all seedlings).

Seedlings (and vegetative individuals) of *C. disticha* and *C. arenaria* could not be discriminated, and it is thus uncertain whether only one or both species were present. Following the conservative approach, they were counted as one taxon.

Of the 57 animals, 10 (17.5%), 12 (21.1%), and 16 (28.1%) carried no seeds in their fur, between their hooves or in their gut, respectively. 23 (40.4%), 15 (26.3%), and 18 (31.6%) carried more than 10 seeds, and 6 (10.5%), 1 (1.8%) and 2 (3.5%) carried more than 100 seeds in their fur, between their hooves or in their gut.

**Figure 2:**
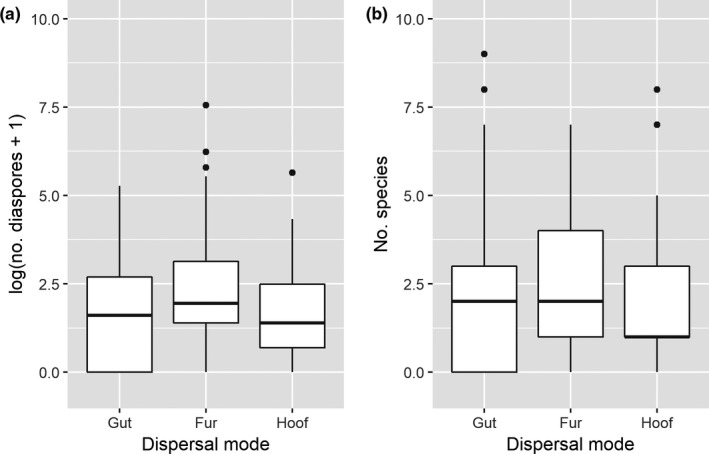
(a) The distribution of number of diaspores from each sampled animal within each dispersal mode (numbers have been log‐transformed). (b) The distribution of number of identified species from each sampled animal within each dispersal mode

Across sampled animals and sampling sites, we found the number of diaspores to peak in fur samples and be lowest in hoof brushings, the differences being significant (*z* = −5.32 – (−41.5), *p*: <.001). In contrast, gut samples did in several cases contain a slightly higher number of species, all comparisons being nonsignificant, however (*z* = −0.023 – (−2.162), *p*: .077 – >.9; Figure 2).

### 3.1 Species composition

The NMDS ordination coordinates (and thus: species composition) were significantly affected by dispersal mode on the first two ordination axes (likelihood‐ratio test of axis 1, 2, and 3, respectively: *L* = 76.888 [*df* = 2, *p* < .001], *L* = 37.478 [*df* = 2, *p* < .001], and *L* = 0:0.661 [*df* = 2, *p* = .7187]; Table 2a–c). ∆AIC > 2 compared to the null model for all three axes.

**Table 2:** Differences in seed species composition between dispersal modes evaluated with linear mixed effects models and with sample coordinates along NMDS ordination axes 1, 2, and 3 as response variables. (a) NMDS axis 1, (b) NMDS axis 2, and (c) NMDS axis 3

Fixed effects				Random effects		
	Estimate	*SE*	*t*‐value	Groups	Variance	*SD*
(a) NMDS axis 1						
Endozoochory (intercept)	−0.919	0.201	−4.561	ID:Site	0.047	0.217
Fur‐epizoochory	1.217	0.123	9.874	Site	0.120	0.347
Hoof‐epizoochory	1.012	0.123	8.233	Residual	0.310	0.557
(b) NMDS axis 2						
Endozoochory (intercept)	0.153	0.294	0.520	ID:Site	0.028	0.167
Fur‐epizoochory	−0.397	0.105	−3.783	Site	0.315	0.561
Hoof‐epizoochory	−0.701	0.105	−6.695	Residual	0.225	0.475
(c) NMDS axis 3						
Endozoochory (intercept)	−0.143	0.189	−0.761	ID:Site	0.042	0.205
Fur‐epizoochory	0.037	0.116	0.316	Site	0.105	0.325
Hoof‐epizoochory	0.093	0.115	0.803	Residual	0.274	0.523

Estimate = variable coefficient. As the predictor variable (Dispersal mode) is factorial, endozoochory is used as baseline and all other coefficients are relative to that.

Abbreviations:* SE*, Standard error, *SD*, Standard deviation.

The endozoochorous species composition (gut) was significantly different from fur‐epizoochorous species composition in two dimensions (post hoc Tukey test, axis 1: *z* = 9.874, *p* < .001; axis 2: *z* = −3.783, *p* < .001; axis 3: *z* = 0.316, *p* = .947).

The endozoochorous species composition was significantly different from hoof‐epizoochorous species composition in two dimensions (post hoc Tukey test, axis 1: *z* = 8.233, *p* < .001; axis 2: *z* = −6.695, *p* < .001; axis 3: *z* = 0.803, *p* = .701).

**Figure 3:**
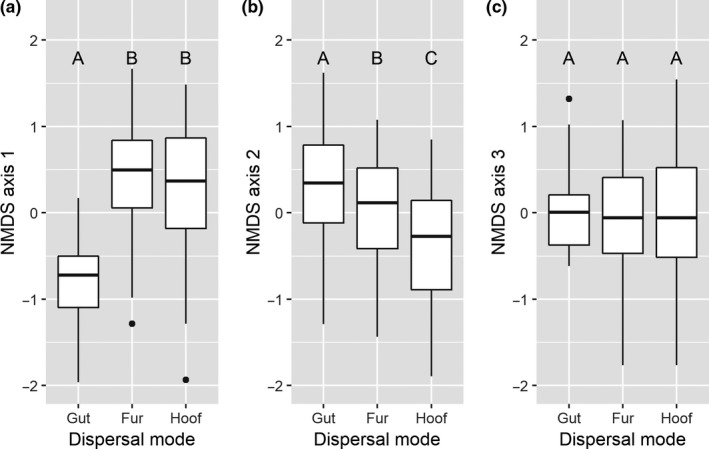
Coordinates of the NMDS ordination for each sample (individual deer) within each dispersal mode. Coordinates along the first axis (NMDS axis 1) (a), second axis (NMDS axis 2) (b) and third axis (NMDS axis 3) (c). The ordination is done based on species composition of each sample. Variation in coordinates between the three dispersal modes indicates significantly different species compositions between the groups. Statistically significant differences in coordinates are indicated with letters (post hoc Tukey test, *p* < .05)

The fur‐epizoochorous species composition was significantly different from hoof‐epizoochorous species composition in one dimension (post hoc Tukey test, axis 1: *z* = −1.734, *p* = .192; axis 2: *z* = −3.015, *p* = .007; axis 3: *z* = 0.506, *p* = .868; Figure 3).

Thus, the species compositions of the three dispersal modes are significantly different, however with some degree of overlap, based on their clustered positions in ordination space. The overlap is larger between the two modes of epizoochory than between endo‐ and epizoochory.

### 3.2 Influence of plant/diaspore characteristics

The conservative approach regarding unidentified diaspores was employed in the LDA.

**Figure 4:**
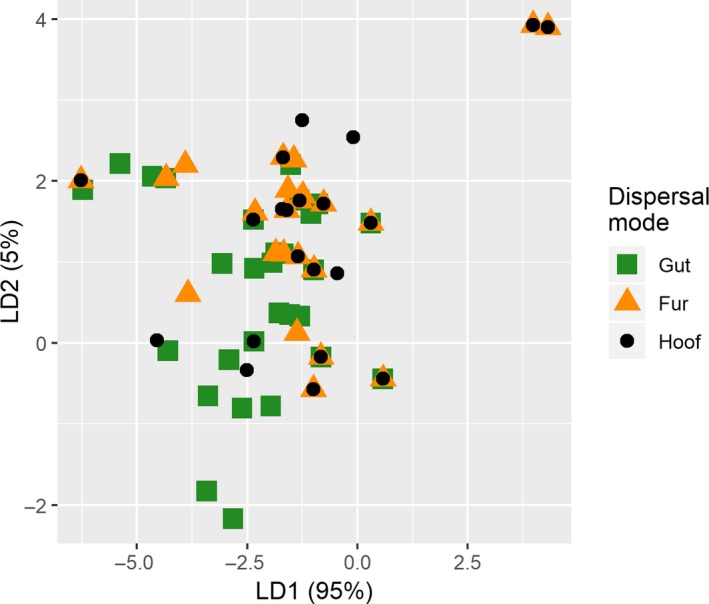
Visualization of separation of the data by the linear discriminant analysis (LDA). The more separated the different dispersal modes, the better the LDA can discriminate between the groups, based on the predictor variables. Percentage figures in parentheses after axis titles indicate the between‐group variance explained by the linear discriminants

The first discriminant function (LD1) achieves 95% of the separation, whereas the second discriminant function (LD2) achieves the remaining 5% (Figure 4). Based on the absolute values of the coefficients in the first discriminant function, the influence of the variables on the separation is in descending order: Releasing height > SNP > Landscape occupancy > Seed shape > Seed mass (Table 3). The relative importance of the variables for the second discriminant function is: Seed weight > Releasing height > Seed shape > SNP > Landscape occupancy.

**Table 3:** Prediction of dispersal mode by seed traits and whole‐plant traits assessed with linear discriminant analysis. The relative importance of the predictor variables can be assessed by comparing the absolute values of the coefficients of the LDA

	LD1	LD2
Log(seed mass)	−0.145	1.033
Log(releasing height)	0.698	0.610
Log(SNP)	0.545	0.038
Seed shape (Vs)	0.172	−0.133
Landscape occupancy	0.418	−0.034

Note

Seed shape = variance in dimensions. Landscape occupancy = proportion of the 25 5 km × 5 km grid cells centered on the sampling site, in which the species is present.

Abbreviation: SNP, Seed number per ramet.

Both endozoochory and fur‐epizoochory are correctly predicted more often than not, which is not the case for hoof‐epizoochory (Table 4). The global misclassification rate of the LDA is 21.2%. The misclassification rate for endozoochory is 27.6%, for fur‐epizoochory 19.5%, and for hoof‐epizoochory 59.1%.

**Table 4:** Dispersal mode predicted for each diaspore by the LDA (columns) versus the observed dispersal mode (rows). The diagonal represents the correct predictions, highlighted in bold. Seeds for which all predictor variables were not available were excluded from the analysis

Observed	Predicted			
	Endozoochory	Fur‐epizoochory	Hoof‐epizoochory	No. observed seeds within dispersal mode
Endozoochory	**210**	188	0	398
Fur‐epizoochory	47	**1,837**	29	1,913
Hoof‐epizoochory	33	258	**20**	311
No. predicted seeds within dispersal mode	290	2,283	49	

Dispersal mode was not dependant on seed appendages (Fisher's exact test, *p* = .22; Table 5).

**Table 5:** Observed species numbers bearing the specified appendage type and Pearson residuals from a *Χ* ^2^ contingency table (observed‐expected/$\sqrt{\text{expected}}$) for each dispersal mode. A positive residual indicated more species with the particular appendage was observed than what was expected from the null hypothesis, a negative residual indicated fewer species with the particular appendage type was observed, compared to the null hypothesis

	Bristly	Hairs	Hooks	None	Other	Winged
Endozoochory						
Observed no. species	3	4	0	26	0	0
Pearson residual	−0.556	0.976	−1.016	0.538	−0.587	−1.173
Fur‐epizoochory						
Observed no. species	5	1	3	21	0	1
Pearson residual	0.500	−0.516	2.000	−0.350	−0.577	−0.289
Hoof‐epizoochory						
Observed no. species	4	1	0	21	1	3
Pearson residual	0.066	−0.483	−0.984	−0.200	1.193	1.505

Due to low numbers of diaspores with appendages, a chi‐squared‐test was performed on a coarser scale, classifying the seeds as “with” or “without” appendages. The difference was nonsignificant as well (*Χ* ^2^ = 1.681, *df* = 2, *p* = .432). Dispersal mode is dependent on habitat association of the mother plant (Fisher's exact test, *p* = .015; Table 6). The likely drivers of this dependence are an overweight of ruderal species compared to the null model (Pearson residual = 2.130), and few woodland species (Pearson residual = −1.661) in the endozoochorous seedlings, few ruderal species compared to the null model (Pearson residual = −1.960) and relatively many woodland species (Pearson residual = 0.985) in the fur‐epizoochorous species. Most of the dispersed species were grassland species.

**Table 6:** Observed species numbers for each habitat association and Pearson residuals from a *Χ* ^2^ contingency table (observed−expected/$\sqrt{\text{expected}}$ ) for each dispersal mode. A positive residual indicated more species with the particular appendage was observed than what was expected from the null hypothesis, a negative residual indicated fewer species with the particular appendage type was observed, compared to the null hypothesis

	Grassland	Ruderal	Wetland	Woodland
Endozoochory				
Observed no. species	15	11	3	3
Pearson residual	0.333	2.130	−0.828	−1.661
Fur‐epizoochory				
Observed no. species	15	1	5	10
Pearson residual	0.456	−1.960	0.154	0.985
Hoof‐epizoochory				
Observed no. species	10	5	6	9
Pearson residual	−0.808	−0.207	0.698	0.714

## 4 DISCUSSION

Our study shows that the species composition of plants seeds dispersed by red deer in Denmark, either through the guts or attached to the fur or hooves, differs significantly, especially between internal (guts) and external (fur and hooves) dispersal. Plant species association with dispersal route could be predicted based on certain seed and whole‐plant traits. Surprisingly, possession of specialized adhesive appendages was not among the predictive traits. More than 50 red deer individuals were investigated for plant diaspores dispersed via gut passage or attachment to fur or hooves. A total of 4,616 diaspores and 958 seedlings from 66 species (minimum) were identified in the epizoochorous (fur and hooves) and endozoochorous (gut) samples, respectively.

Species composition of the three dispersal modes generally overlapped, but with a significant proportion of species being associated with a certain dispersal mode. The number of plant species was similar between dispersal modes. However, these numbers are far from directly comparable; the quantity of diaspores in half a total fleece probably represents much more extended time of vegetation–animal interaction than does the quantity of diaspores in 100 g of feces from the animal's rectum. The number of individual animals investigated was much higher than in any previous study of red deer epizoochory, but the amount of feces collected lower than in some previous studies of red deer endozoochory. This reflects the fact that collection of feces left behind is relatively easy compared to investigating fur or hooves, apparently leading to the false impression that endozoochory is a more important seed dispersal route than is epizoochory. Comparisons of seed load and species numbers between dispersal modes should be done cautiously, and we draw no further conclusions on the importance of either dispersal mode relative to the others, regarding seed or species numbers. However, the reported comparison of the dispersal‐related traits possessed by plants preferentially dispersed through one route more than the others is much less sensitive to the uneven sampling intensity.

The fur‐borne seed load of half the fleece of an animal ranged from 0 to 1,912 seeds, and the hoof‐borne seed load from all four hooves together ranged from 0 to 282 seeds. The most abundant species for these two modes of epizoochory were *J. effusus*, with 76% of all seeds in the fur, and 49% of all seed from the hooves. Many of the *J. effusus* seeds were found inside intact capsules and even parts of infructescences, which partly accounts for the high seed load of this particular species. This overweight of *Juncus* has not been found in other studies (e.g., Couvreur et al. (2005) [donkeys] and Heinken and Raudnitschka (2002) [roe deer and wild boar]), but was to be expected given the abundance of *J. effusus* in the study areas and the profuse fecundity of the species. In addition, common and locally abundant grasses were well represented in the fur samples, in particular *Molinia caerulea*, *A. capillaris*, and *Deschampsia cespitosa*, which is well aligned with previous findings from Germany (sheep), Denmark (dog), and France (red deer, wild boar and roe deer; Fischer et al., 1996; Graae, 2002; Picard et al., 2016). Seeds of genus *Juncus* were abundant in hoof samples and probably also belong to *J. effusus*, although they occurred as individual seeds and could not always be identified safely to the species level. Further species frequently found on hooves were *Betula pubescens*, *D. cespitosa*, and *A. capillaris*. For *Betula*, having winged seeds borne in tree canopies, epizoochory via hoof attachment must represent secondary seed dispersal (Picard & Baltzinger, 2012). Abundance of *Betula* and grass seeds was also found by Heinken and Raudnitschka (2002) and Picard and Baltzinger (2012).

Seedling emergence occurred in 84% of the gut samples, with an average density of 108.5 seedlings per 100 g dry mass feces. This density is in the range found by other studies, for example, 70 seedlings per 100 g (England, red deer/fallow deer; Panter & Dolman, 2012) and 642 seedlings per 100 g (Germany, red deer; von Oheimb et al., 2005). The most abundant endozoochorous species were *A. capillaris*, a dominant grass, *Juncus bufonius*, a fecund ruderal species, and *Urtica dioica*, a tall, grazing resistant forb. This is well aligned with previous findings, for example, Iravani et al. (2011), Jaroszewicz, Pirożnikow, and Sondej (2013), Karimi et al. (2018), Lepková et al. (2018), and Panter and Dolman (2012).

The numerical dominance of small‐seeded species such as *Juncus* spp. in all three dispersal modes can be attributed to an effect of high seed number per plant, rather than an effect of small size per se (Bruun & Poschlod, 2006). However, since no direct data on seed abundance on the landscape scale were available, it remains intractable to discriminate safely between the two effects.

The differences in species composition were evaluated by assessing differences in position along the axes in an NMDS ordination space. The axes themselves have no ecological meaning, but the overlap (or lack thereof) of the sample point clouds do. As the ordination was performed on a species‐by‐sample matrix, samples with similar species composition will be placed closer together in the ordination space and vice versa. The overlap in composition of species transported by the three dispersal modes suggests that most plant diaspores are picked up by deer as blind passengers in proportion to their abundance on the landscape scale. However, the NMDS results showed significant differences in species compositions between endozoochory and epizoochory in particular. This suggests that deer to some extent either selectively ingest seeds with infructescences or that some plants present seeds within attractive foliage (Janzen, 1984). Couvreur et al. (2005) proposed that endozoochory and epizoochory are complementary rather than additive dispersal processes regarding dispersed species, which is somewhat shown in the review by Baltzinger et al. (2019) as well. Interestingly, many of the species separating endozoochory from epizoochory in this study are species associated with ruderal habitats, probably having more palatable foliage and being selected by the animals (Cates & Orians, 1975).

Dispersal mode was correctly predicted from plant and diaspore traits more often than not, but nonetheless with much uncertainty. The three most influential variables affecting the predicted dispersal mode (LD1, 95% of variance between classes explained) were, in descending order, releasing height, seed production, and landscape occupancy. Hoof‐epizoochory was falsely predicted more often than not. This supports the notion that dispersal via hoof attachment is merely accidental, picking up seeds, which have fallen to the ground or already been incorporating into the soil seedbank. Secondary dispersal by deer may nonetheless account for plant species colonizing otherwise inaccessible habitat.

The multivariate analysis of both species composition and plant and seed traits suggests that species' probability of fur‐borne dispersal is more deterministic than hoof‐borne; or, at the very least, that deer behavior for resting couchant and wallowing influences species differentially. Overall, however, single‐seed adaptations, such as hooks and bristles, have a lot less predictive power than held traditionally (Albert, Auffret, et al., 2015; Albert, Mårell, et al., 2015). Vector behavior will affect both diaspore attachment, for example, through selective foraging, and through grooming behavior, as was shown by Liehrmann et al. (2018). The first point is especially true for an intermediate mixed feeder, such as red deer (Baltzinger et al., 2019; Karimi et al., 2018; Picard et al., 2016).

The present study has demonstrated that red deer hold the potential to transport appreciable amounts of plant diaspores, both in the fur, on the hooves and through the intestinal tract. From the present study, as for most studies of seed dispersal, it remains unknown if the demonstrable movement of seed has a significant effect on plant population dynamics, in particular alleviating dispersal‐limited colonization of empty, but suitable habitat. Plant species dominant on the landscape scale are more likely to become transported, but less likely to end up in vacant biotopes. Habitat specialists would have higher potential of gaining in site occupancy from seed dispersal, if only being picked up by the animals and released again in a suitable site. From a conservation perspective, that would be relevant to forest specialists in a deforested landscape matrix or to wetland plants in a well‐drained landscape matrix. However, the habitat specialists that mostly seemed to benefit from deer zoochory in the present study were ruderal species and grassland species. This may reflect dispersal of diaspores from sites disturbed by the deer themselves or dispersal of diaspores from arable field, visited by red deer for nutritious forage, and released again in more natural vegetation. In any case, this result is aligned with findings elsewhere that forest plant species are dispersed less between forest fragments, than are species from the arable landscape matrix dispersed into forest fragments (Panter & Dolman, 2012; Picard et al., 2016).

Considering the increasing red deer populations in Denmark and in Europe in general, the impact of deer‐mediated zoochory is likely to grow. This is expected to increase the potential for haphazard dispersal of less abundant plant species and nonruderal habitat specialists. However, predictive modeling of plant colonization following defaecation or detachment is quite intractable.

## CONFLICT OF INTEREST

The authors declare no competing interests.

## AUTHOR CONTRIBUTIONS

HHB and TKP conceived the idea and designed the study. TKP undertook the fieldwork, laboratory work, made the statistical analysis, and wrote the first draft.

## Data Availability

Raw data available at FigShare, https://doi.org/10.6084/m9.figshare.7982483

## APPENDIX 1

**Table A1:** Species list of all identified species from the fur and hoof samples. Numbers are specified for each of the locations, Jægersborg Dyrehave (*n* = 10), Lille Vildmose (*n* = 21), Oksbøl (*n* = 22), and Torbenfeldt Manor (*n* = 4)

Species	Site	Fur	Subtotal	Total
		Hoof		
*Agrostis canina*	Oksbøl	5	5	5
		–		
*Agrostis capillaris*	Jægersborg Dyrehave	165	179	219
		14		
	Lille Vildmose	4	14	
		10		
	Oksbøl	7	16	
		9		
	Torbenfeldt Manor	8	10	
		2		
*Aira praecox*	Oksbøl	1	1	1
		–		
*Alnus glutinosa*	Jægersborg Dyrehave	–	2	6
		2		
	Lille Vildmose	4	4	
		–		
*Aphanes inexpectata*	Oksbøl	1	1	1
		–		
*Asteraceae* *	Lille Vildmose	–	1	1
		1		
*Betula pubescens*	Lille Vildmose	39	88	100
		49		
	Oksbøl	11	12	
		1		
*Betula* spp.**	Jægersborg Dyrehave	1	2	8
		1		
	Lille Vildmose	–	3	
		3		
	Oksbøl	1	1	
		–		
	Torbenfeldt Manor	1	2	
		1		
*Brachypodium sylvaticum*	Jægersborg Dyrehave	2	2	2
		–		
*Calamagrostis epigejos*	Jægersborg Dyrehave	–	1	78
		1		
	Lille Vildmose	49	77	
		28		
*Calluna vulgaris*	Oksbøl	27	43	43
		16		
*Carduus*/*Cirsium* spp.	Torbenfeldt Manor	1	2	2
		1		
*Carex arenaria*	Oksbøl	16	16	16
		–		
*Carex arenaria*/*ovalis* ***	Lille Vildmose	4	4	4
		–		
*Carex echinata*	Oksbøl	1	1	1
		–		
*Carex ovalis*	Lille Vildmose	2	3	5
		1		
	Oksbøl	2	2	
		–		
*Carex polyphylla*	Jægersborg Dyrehave	6	20	20
		14		
*Carex* spp.**	Lille Vildmose	2	3	6
		1		
	Oksbøl	1	3	
		2		
*Cerastium fontanum*	Oksbøl	–	2	2
		2		
*Circaea lutetiana*	Jægersborg Dyrehave	4	4	4
		–		
*Dactylis glomerata* ssp.* glomerata*	Jægersborg Dyrehave	1	1	2
		–		
	Lille Vildmose	–	1	
		1		
*Deschampsia cespitosa*	Jægersborg Dyrehave	147	177	211
		30		
	Lille Vildmose	24	34	
		10		
*Deschampsia flexuosa*	Lille Vildmose	5	22	28
		17		
	Oksbøl	6	6	
		–		
*Epilobium adenocaulon*/*ciliatum* ***	Lille Vildmose	–	1	1
		1		
*Epilobium obscurum*	Lille Vildmose	–	1	1
		1		
*Epilobium palustre*	Lille Vildmose	–	1	1
		1		
*Galeopsis ladanum*	Torbenfeldt Manor	–	3	3
		3		
*Galium odoratum*	Torbenfeldt Manor	1	1	1
		–		
*Geum urbanum*	Jægersborg Dyrehave	4	4	6
		–		
	Torbenfeldt Manor	2	2	
		–		
*Holcus lanatus*	Lille Vildmose	3	3	3
		–		
*Juncus effusus*	Jægersborg Dyrehave	321	560	3,308
		239		
	Lille Vildmose	2,511	2,631	
		120		
	Oksbøl	117	117	
		–		
*Juncus* spp.**	Jægersborg Dyrehave	5	6	89
		1		
	Lille Vildmose	17	61	
		44		
	Oksbøl	2	8	
		6		
	Torbenfeldt Manor	14	14	
		–		
*Lolium perenne*	Lille Vildmose	3	3	3
		–		
*Molinia caerulea*	Lille Vildmose	220	243	304
		23		
	Oksbøl	51	61	
		10		
*Myosotis laxa*	Lille Vildmose	1	1	1
		–		
*Oxalis acetosella*	Lille Vildmose	–	2	2
		2		
*Pastinaca sativa*	Torbenfeldt Manor	–	3	3
		3		
*Persicaria minor*	Lille Vildmose	2	2	2
		–		
*Phalaris arundinacea*	Jægersborg Dyrehave	–	1	1
		1		
*Picea glauca*	Oksbøl	–	1	1
		1		
*Poa annua*	Lille Vildmose	–	11	11
		11		
*Poa trivialis*	Lille Vildmose	1	1	1
		–		
*Poa* sp.****	Lille Vildmose	–	6	6
		6		
*Polygonum aviculare*	Lille Vildmose	1	3	5
		2		
	Oksbøl	2	2	
		–		
*Ranunculus flammula*	Lille Vildmose	1	2	2
		1		
*Rubus idaeus*	Torbenfeldt Manor	–	1	1
		1		
*Rumex acetosella*	Lille Vildmose	1	2	3
		1		
	Oksbøl	–	1	
		1		
*Rumex sanguineus*	Jægersborg Dyrehave	43	60	60
		17		
*Trichophorum cespitosum*	Lille Vildmose	–	1	1
		1		
*Urtica dioica*	Jægersborg Dyrehave	–	1	31
		1		
	Lille Vildmose	–	1	
		1		
	Torbenfeldt Manor	12	29	
		17		
Minimum no. of species for total epizoochory: 46 Minimum no. of species for fur‐epizoochory: 32 Minimum no. of species for hoof‐epizoochory: 32 Maximum no. of species: 49				4,616

Note

“Minimum no. species” indicates a conservative count: All diaspores not identified to species level are assumed to belong to an already identified species, unless this was obviously not the case. “Maximum no. species” indicates the species number, if diaspores not identified to species level belongs to a new species.

* The diaspore was too damaged to make a more specific identification than to family level.

** The diaspores could not be identified further than to genus level due to damage or other missing characters.

*** Species/genera could not be told apart.

**** Not belonging to the two identified *Poa*‐species, but species could not be determined exactly.

**Table A2:** Species list of all identified species from the gut samples. Numbers are specified for each of the locations, Jægersborg Dyrehave (*n* = 10), Lille Vildmose (*n* = 21), Oksbøl (*n* = 16), and Torbenfeldt Manor (*n* = 3)

Species	Site		Total
*Agrostis capillaris*	Jægersborg Dyrehave	123	182
	Lille Vildmose	44	
	Oksbøl	14	
	Torbenfeldt Manor	1	
*Agrostis gigantea*	Jægersborg Dyrehave	3	25
	Lille Vildmose	15	
	Oksbøl	7	
*Agrostis stolonifera*	Lille Vildmose	9	9
*Calamagrostis epigejos*	Lille Vildmose	4	4
*Carex arenaria*/*disticha* *	Lille Vildmose	69	75
	Oksbøl	6	
*Carex sylvatica*	Oksbøl	1	1
*Cerastium fontanum*	Jægersborg Dyrehave	1	4
	Lille Vildmose	1	
	Oksbøl	2	
*Dactylis glomerata*	Jægersborg Dyrehave	4	13
	Lille Vildmose	9	
*Deschampsia cespitosa*	Lille Vildmose	6	6
*Deschampsia flexuosa*	Lille Vildmose	3	3
*Festuca ovina*	Lille Vildmose	1	1
*Festuca pratensis*	Jægersborg Dyrehave	25	25
*Galium palustre*	Lille Vildmose	2	2
*Gnaphalium uliginosum*	Lille Vildmose	2	2
*Holcus lanatus*	Lille Vildmose	1	1
*Juncus articulaus*	Lille Vildmose	91	92
	Oksbøl	1	
*Juncus bufonius*	Jægersborg Dyrehave	61	110
	Lille Vildmose	18	
	Oksbøl	31	
*Juncus effusus*	Jægersborg Dyrehave	15	54
	Lille Vildmose	37	
	Oksbøl	2	
*Juncus tenuis*	Jægersborg Dyrehave	17	17
*Leontodon autumnalis*	Lille Vildmose	1	1
*Molinia caerulea*	Lille Vildmose	1	1
*Plantago major* ssp. *major*	Jægersborg Dyrehave	12	16
	Lille Vildmose	4	
*Poa annua*	Jægersborg Dyrehave	6	6
*Poa pratensis*	Jægersborg Dyrehave	63	65
	Lille Vildmose	2	
*Polygonum aviculare*	Jægersborg Dyrehave	1	1
*Rumex acetosella*	Lille Vildmose	16	16
*Sagina procumbens*	Lille Vildmose	29	29
*Sisymbrium officinale*	Jægersborg Dyrehave	1	1
*Spergularia rubra*	Jægersborg Dyrehave	1	35
	Lille Vildmose	1	
	Oksbøl	33	
*Stellaria graminea*	Lille Vildmose	6	6
*Urtica dioica*	Jægersborg Dyrehave	105	107
	Torbenfeldt Manor	2	
*Veronica beccabunga*	Lille Vildmose	2	2
*Veronica officinalis*	Lille Vildmose	46	46
Total number of species: 33			958

Note

* Species could not be told apart.
